# The gene SMART study: method, study design, and preliminary findings

**DOI:** 10.1186/s12864-017-4186-4

**Published:** 2017-11-14

**Authors:** Xu Yan, Nir Eynon, Ioannis D. Papadimitriou, Jujiao Kuang, Fiona Munson, Oren Tirosh, Lannie O’Keefe, Lyn R. Griffiths, Kevin J. Ashton, Nuala Byrne, Yannis P. Pitsiladis, David J. Bishop

**Affiliations:** 1 Institute of Sport, Exercise and Active Living (ISEAL), College of Sport and Exercise Science, Victoria University, PO Box 14428, Melbourne, VIC 8001 Australia; 2 College of Health and Biomedicine, Victoria University, Melbourne, Australia; 3 Murdoch Childrens Research Institute, Royal Children’s Hospital, Melbourne, Australia; 4 School of Health Sciences, Swinburne University of Technology, Melbourne, Australia; 5 Institute of Health and Biomedical Innovation (IHBI), Genomics Research Centre, Queensland University of Technology, Brisbane, Australia; 6 Bond Institute of Health and Sport (BIHS), Bond University, Gold Coast, Australia; 7 FIMS Reference Collaborating Centre of Sports Medicine for Anti-Doping Research, University of Brighton, Eastbourne, UK; 8 School of Medical and Health Sciences, Edith Cowan University, Joondalup, Australia

**Keywords:** Genetic variants, Skeletal muscle, Training

## Abstract

The gene SMART (genes and the Skeletal Muscle Adaptive Response to Training) Study aims to identify genetic variants that predict the response to both a single session of High-Intensity Interval Exercise (HIIE) and to four weeks of High-Intensity Interval Training (HIIT). While the training and testing centre is located at Victoria University, Melbourne, three other centres have been launched at Bond University, Queensland University of Technology, Australia, and the University of Brighton, UK. Currently 39 participants have already completed the study and the overall aim is to recruit 200 moderately-trained, healthy Caucasians participants (all males 18–45 y, BMI < 30). Participants will undergo exercise testing and exercise training by an identical exercise program. Dietary habits will be assessed by questionnaire and dietitian consultation. Activity history is assessed by questionnaire and current activity level is assessed by an activity monitor. Skeletal muscle biopsies and blood samples will be collected before, immediately after and 3 h post HIIE, with the fourth resting biopsy and blood sample taken after four weeks of supervised HIIT (3 training sessions per week). Each session consists of eight to fourteen 2-min intervals performed at the pre-training lactate threshold (LT) power plus 40 to 70% of the difference between pre-training lactate threshold (LT) and peak aerobic power (W_peak_). A number of muscle and blood analyses will be performed, including (but not limited to) genotyping, mitochondrial respiration, transcriptomics, protein expression analyses, and enzyme activity. The participants serve as their own controls. Even though the gene SMART study is tightly controlled, our preliminary findings still indicate considerable individual variability in both performance (in-vivo) and muscle (in-situ) adaptations to similar training. More participants are required to allow us to better investigate potential underlying genetic and molecular mechanisms responsible for this individual variability.

## Background

### Individual variability in the response to similar exercise training

Despite the proven health and performance benefits of exercise training, it is clear there is considerable individual variability in the response to similar exercise training [1–4]. This means some people are ‘low/medium responders’ (with limited improvements following exercise training), while others are ‘high-responders’ [5]. A high inter-individual variability in the response to similar physical exercise is consistently reported in training studies, even within homogenous groups of previously untrained subjects and after fully compliant and supervised training [6, 7]. For example, in the pioneering Health, Risk factors, Training and Genetics (HERITAGE) study, changes in aerobic capacity were observed to vary markedly in a group of sedentary adults after similar exercise training [1]. Recently, we have also shown large individual variability for changes in skeletal muscle mitochondrial function (respiration) in response to high-intensity exercise training [8]. Identifying and understanding the molecular pathways contributing to the individual response to exercise training is challenging, but has exciting potential implications for “personal medicine” and the future development of individualised exercise training programs [9]. While exercise training is an important non-pharmacological intervention to improve health, and to reduce the risk for many chronic diseases, it is anticipated that personalising training using not only environmental factors (e.g., specific diet and training etc.), but also biological markers (i.e., genomics, transcriptomics, proteomics, and metabolomics) will have important health and economic ramifications by ensuring that specific exercise interventions are prescribed so as to attain the greatest benefit.

### Genetic basis of individual response to exercise training

The underlying aetiology for this large variation in the training response remains to be discovered. However, different lines of research indicate there is a strong genetic component. Studies in the 1980s, involving twins and siblings, have demonstrated that variance in maximal oxygen uptake ($\overset{\cdotp }{\mathrm{V}}{\mathrm{O}}_{2\max }$) and the lactate threshold (LT) was smaller within monozygotic than within dizygotic twins and brothers of the same sibship [10, 11]. Furthermore, even though there were large individual differences in response to a 3-month exercise intervention with controlled energy intake, participants with the same genotype (i.e. monozygotic twins) were more alike in responses than participants with different genotypes (between different pairs of monozygotic twins), particularly for changes in body fat, body weight, and abdominal visceral fat [12]. Adaptations in $\overset{\cdotp }{\mathrm{V}}{\mathrm{O}}_{2\max }$ and endurance performance after 15 weeks of exercise training were also more similar (>4.6 and >9.7 times more similar respectively) within the monozygotic twin pair than between different pairs monozygotic twins [11]. Similarly, changes in muscle enzyme activities after training, such as malate dehydrogenase and oxoglutarate dehydrogenase, were partially genetically dependent, with more similar adaptations within the monozygotic twin pairs than between different pairs of twins during the last 8 weeks of training [11].

The most comprehensive data, thus far, concerning the genetic contribution of the responsiveness to a standardised exercise training program, arises from the HERITAGE study with the calculated heritability of training adaptations in $\overset{\cdotp }{\mathrm{V}}{\mathrm{O}}_{2\max }$ reported to range from 45% to 50% [13]. To further explore the potential underlying genetic component, the candidate gene approach was first employed. More than 100 candidate gene variants have subsequently been reported to be associated with the response to exercise training [14–16]; however, most of these variant have not been replicated in other studies and it is likely that some of them are false positives and not truly associated with training responses. Developments in microarray-based, high-throughput technologies have allowed researchers to move beyond the candidate gene approach to unbiased analysis of thousands of common Single-Nucleotide Polymorphisms (SNPs) simultaneously [13]. However, to date, only a few genome-wide association (GWA) trainability studies have been published, all using $\overset{\cdotp }{\mathrm{V}}{\mathrm{O}}_{2\max }$ as a response trait, and all have arisen from the HERITAGE cohort [16–18].

In the first report, microarray was used to identify genes associated with the $\overset{\cdotp }{\mathrm{V}}{\mathrm{O}}_{2\max }$ training response, based on global skeletal muscle gene expression profiling and DNA markers of 24 participants [17]. A total of 29 transcripts were strongly associated with the gains in $\overset{\cdotp }{\mathrm{V}}{\mathrm{O}}_{2\max }$, with 11 SNPs explaining approximately 23% of the variance in the $\overset{\cdotp }{\mathrm{V}}{\mathrm{O}}_{2\max }$ training response [17]. The second GWA report, with 473 participants from the HERITAGE study, analysed more than 320,000 SNPs [16]. A total of 39 different SNPs were associated with the $\overset{\cdotp }{\mathrm{V}}{\mathrm{O}}_{2\max }$ training response, with the strongest evidence of association observed in the first intron of the acyl-coA synthetase longchain family member 1 (*ACSL1*) gene [16]. Nine SNPs each explained at least 2% of the variance, while 7 contributed between 1 and 2% each [16]. This study showed that 21 SNPs accounted for 49% of the variance in $\overset{\cdotp }{\mathrm{V}}{\mathrm{O}}_{2\max }$ trainability, a value comparable to the heritability estimate of 47% in $\overset{\cdotp }{\mathrm{V}}{\mathrm{O}}_{2\max }$ trainability reported previously in the HERITAGE study [1]. The third study used a system biology-based approach to predict $\overset{\cdotp }{\mathrm{V}}{\mathrm{O}}_{2\max }$ training response, through considering multiple DNA sequence variants [18]. The study retested the 21 SNPs previously published, by mapping nearly 2.5 million SNPs strictly based on their location within a 20-kb window on either side of a gene. Through this it has been confirmed the SNP in the ACSL1 gene (rs6552828) contributed to about 6% of the training response of $\overset{\cdotp }{\mathrm{V}}{\mathrm{O}}_{2\max }$, as well as SNPs in the other three genes CAMTA1, BIRC7, and CD44 [18]. Five genes did not overlap, and 12 of the 21 genes could not be compared because the genes were mapped more than 20 kb away from the SNPs [18].

### Gaps in literature

Despite advances in the field of Exercise Genomics, several limitations in current training studies exist. First, the participants in the majority of training-studies are not rigorously selected and are not tightly-controlled (i.e., large differences in base-line physical activity-level, nutrition status, sex, and ethnicity). Second, the majority of training studies into the effect of genes on exercise have not provided any proposed cellular or molecular mechanisms to explain the individual variation in the training response. The reason is that these studies are mainly based on physiological phenotypes, such as $\overset{\cdotp }{\mathrm{V}}{\mathrm{O}}_{2\max }$, and have not collected muscle and/or blood samples pre- and post-training. Lastly, many of the training studies have a sample size of between 10 to 30 participants, with a potential large variation in the participant’s base-line physical activity level, and hence relatively small effects of a target gene. Consequently, the Genomic, Transcriptomic, Proteomic, and Metabolomic (OMICS) profile of either low or high responders to exercise training remains poorly characterised.

### Significance and aim of the gene SMART (genes and the skeletal muscle adaptive response to training) study

The gene SMART study, a part of the recently established ATHLOM Consortium [19], is a multi-centre (Victoria University, Australia; Bond University, Australia; Queensland University of Technology, Australian; and The University of Brighton, UK), tightly-controlled, exercise training study (please see “Study Design and Sampling” section). The overarching aim of this multi-centre study is to use a system biology approach (with current state-of-the-art technology) to identify the genomic (gene variants), transcriptomic (gene expression profile), metabolomics (enzyme activities), and proteomic (proteins abundance) factors predicting the response to both a single session of high-intensity interval exercise (HIIE) and to 4 weeks of high-intensity interval training (HIIT), in a relatively-large group (*N* = 200) of participants. It is anticipated that this research will provide significant new information on the biological basis of adaptation to exercise training, and may also have implications for talent identification and the training of elite athletes.

## Study design and sampling

### Study overview

**Fig. 1:**
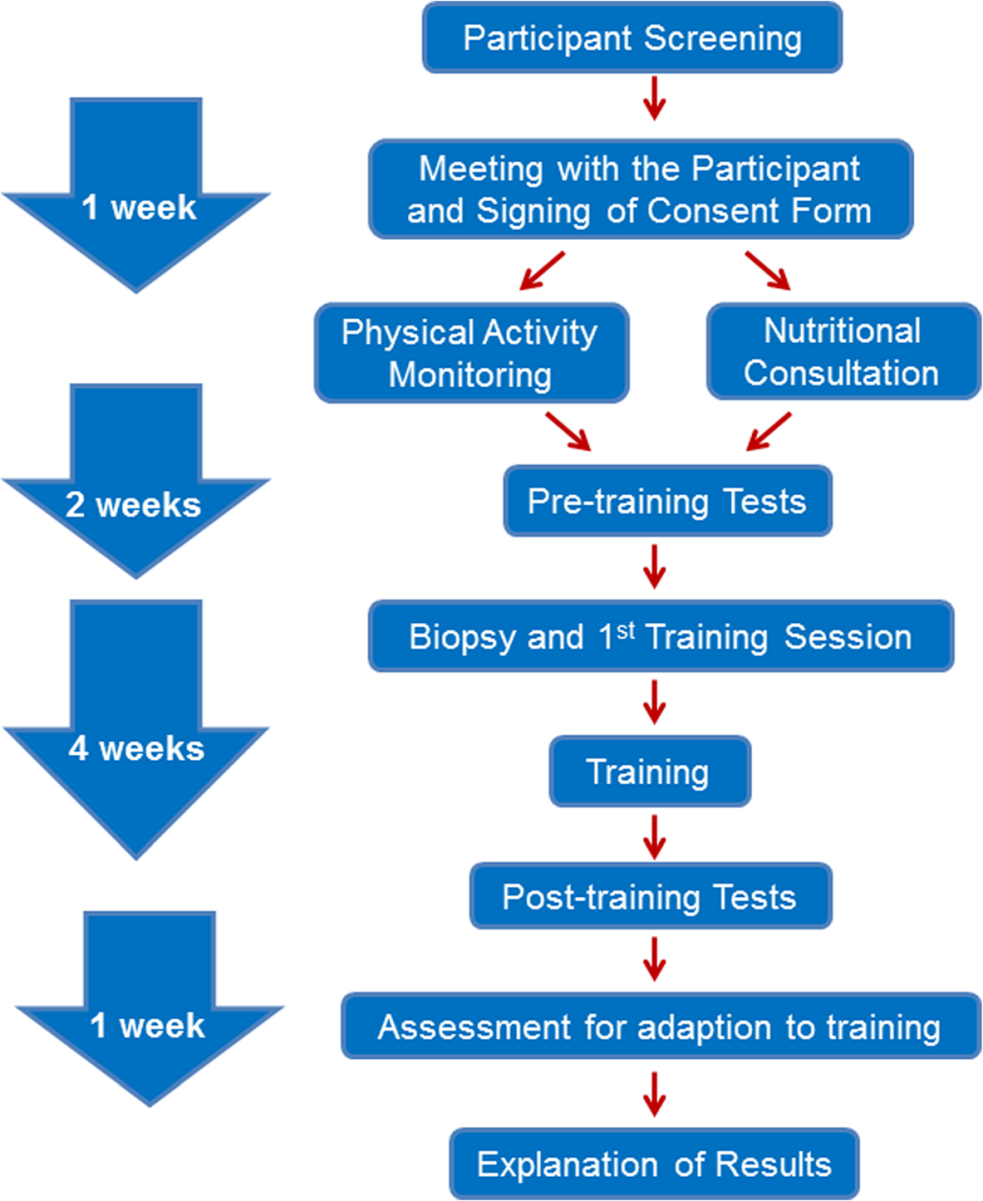
Study flow chart with timeline

**Fig. 2:**
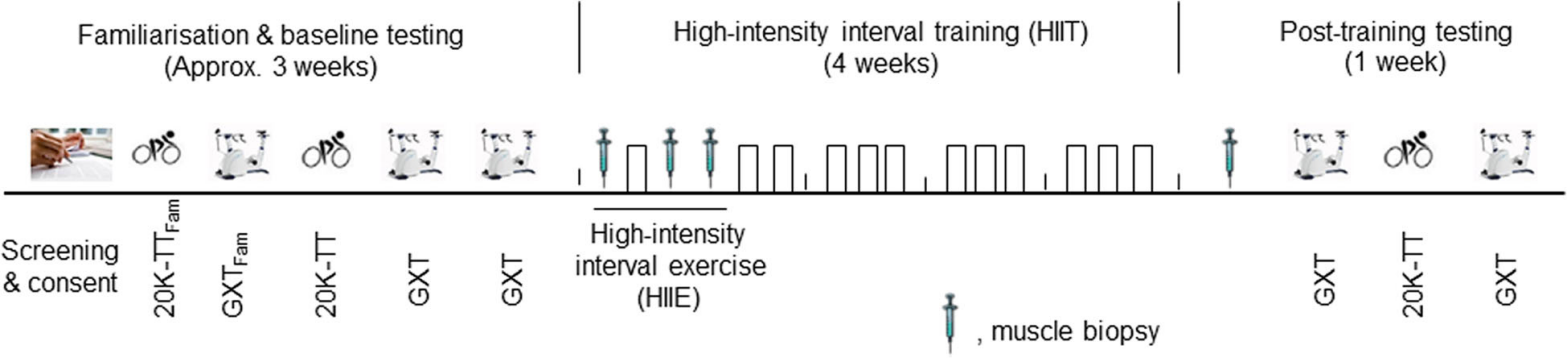
Overview of exercise testing and training. Fam, familiarization; 20 km-TT, 20 km cycle time trial; GXT, graded exercise test to exhaustion; HIIE, high-intensity interval exercise

A study flow chart is shown in Fig. 1. Briefly, potential participants will first express their interest online, and will then be contacted by a research coordinator. If the potential participant satisfies all of the inclusion criteria (please see ‘Participants’ section for the study criteria), they will be scheduled to meet with one of the research team members to receive an in-depth explanation of the study design, as well as an explanation on the benefits and the potential risks of participating in this study. At that time, they will be asked to carefully read and complete a consent form and risk assessment questionnaires. The participant will then be given an activity monitor and a data sheet to monitor their physical activity for 1 week. During this week they will also be scheduled to meet a dietitian, who is a member of our team, and who will provide nutritional consultation for the length of the study. The participants will commence their base-line (pre-training) exercise tests the following week, which will take 2 weeks to complete (Fig. 2). Following the final base-line testing, and 48 h prior to the pre-training resting muscle biopsy, participants will be given a 48-h standardised diet. Two days after the first training session (which includes the resting biopsy as well as 2 post-exercise biopsies) the participants will commence the remaining 11 sessions of HIIT. The participant will repeat the 48-h standardised diet before the post-training resting muscle biopsy. Following the post-training biopsy, the participant will be allowed to recover for 48–72 h and then repeat the pre-training exercise testing to evaluate their training response. Finally, to maximise the participant’s benefits, the exercise training and testing results will be explained to the participant by a senior member of the research team.

### Participants

Two hundred moderately-trained ($\overset{\cdotp }{\mathrm{V}}{\mathrm{O}}_{2\mathrm{peak}}$ 35–60 mL·min^−1^·kg^−1^) men, aged 18 to 45 y, from the student and staff populations of the participating universities and the local communities, will be recruited for the study. For the purpose of genotyping, and to avoid genetic skew, we will ensure that all participants are unrelated Caucasians for ≥3 generations using a written questionnaire and ancestry genomic markers. Participants will have a Body Mass Index (BMI) between 20 and 30 kg·m^−2^ and a body fat percentage < 25% [20, 21].

Recruitment of eligible volunteers is based on extensive publicity and a combination of campus advertisements, newspaper advertisements, radio and television exposures, and community contacts. A university website has also been developed at Victoria University (www.vu.edu.au/speed-gene). A research coordinator has been hired to deal with volunteer communications, and testing & training schedules. Potential participants will initially be screened on the phone, followed by a more extensive screening done at the research centre. The study has been approved by the human ethics committee of each participating institution and written informed consent will be obtained from each participant.

A detailed medical history will be assessed by questionnaire. Participants will be excluded from the study if they have a past history of the following medical conditions: definite or possible coronary heart disease, significant chronic or recurrent respiratory condition, significant neuromuscular, major musculoskeletal problems interfering with ability to cycle, uncontrolled endocrine and metabolic disorders or diabetes requiring insulin and other therapies.

#### Sample size

The proposed sample size (200 participants) is designed to yield enough power for hypothesis testing and to obtain reliable results. The complexity of this study makes it impossible for a single centre to complete all the analysis, which is why three out of four centres (Bond University, Australia; University of Brighton, UK and Queensland University of Technology, Australia) will serve as an ‘OMIC’ centre to perform genomic, transcriptomic, and metabolomic analyses. Similar to the HERITAGE study [22], we have included multiple criteria for inclusion and exclusion. However, we are well aware that heterogeneity among the four centres can be a potential limitation. Before we combine the data together, we will first test the within-centre and among-centre differences.

## Experimental overview

### Pre-training physical activity monitoring

To control for potential differences in habitual physical activity between participants, 1 week prior to commencing the study, we will monitor the participant’s activity level for seven consecutive days. Monitoring participants’ activity level will be performed using an ActiGraph GT3X+ device (ActiGraph LLC, Fort Walton Beach, FL, USA). The GT3X+ activity monitor provides objective measurements of human activity, and has been used in many research [23, 24] and clinical applications [25, 26]. The device includes a micro-electro-mechanical system based tri-axis accelerometer sensor that provides measures of acceleration in three axes, with a composite measure called the vector magnitude (VM = √(×2 + y2 + z2)). The accelerometer has ±6 g range with a sampling rate ranging from 30 Hz to 100 Hz (user selectable) storing the raw, non-filtered/accumulated data, in the units of gravity. The device can sample continuously for between 24 and 32 days depending on the selected sampling frequency.

The ActiGraph GT3X+ device can be threaded onto an elastic belt and worn either over or under clothing, whichever is most comfortable for the participant. The device is positioned snugly enough against the body so that it cannot flop around. The device is worn with the elastic belt fastened around the waist over the right hip bone all day while the participant is awake. The only times the device can be removed is when the participant goes to bed at night, or if the unit would become completely wet (e.g., swimming and showering). A daily diary will assist in monitoring when the device is being removed, and to account for any water-based activities.

The acceleration data will be downloaded from the device and processed over a user-specified time sampling interval. Energy expenditure and activity intensity will be calculated using algorithms that have been derived by independent researchers from around the world [27, 28]. Cutpoints will be used according to Freedson et al. [27], to calculate METs (metabolic rate) and level of activity. METs measure will be incorporated as a covariate, and level of activity will be used to categorise participants into groups; Light, Moderate, Vigorous, and Very Vigorous. The collected data will be incorporated into the data analysis, and used as a covariate in subsequent statistical analyses.

### Nutrition consultation

Each participant will be provided with individualised, pre-packaged meals for the 48 h prior to the first and last resting muscle biopsies. The energy content of the provided meals will be calculated using the Mifflin St-Jeor equation and each participant’s body mass (BM), height and age [29]. The Foodworks *(Xyris)* nutritional data base will be used to determine the nutritional components of the packaged meals and to ensure all nutritional requirements will be met with the diet. The content of the diets are constructed based on the current National Health and Medical Research Council (NHMRC) guidelines. To ensure adequate access to carbohydrate energy stores during each training session, participants will be asked to consume a pre-packaged training meal of high glucose food items (1.5 g·kg^−1^ BM) 2 h prior to the commencement of each training and testing session, according to suggestions from the Australian Institute of Sport (AIS) [30]. Participants will be provided with a post-training and post-testing meal consisting of protein (0.3 g·kg^−1^ BM) and carbohydrates (0.3 g·kg^−1^ BM) [31]. Participants will also be asked to refrain from alcohol and caffeine during the dietary control period, which is 48 h prior to each resting biopsy. Outside of the dietary-control period they will continue with their normal exercise and dietary habits.

### Performance tests

Prior to the start of the High-Intensity Interval Training (HIIT) phase, all participants will complete familiarisation and baseline testing (see Fig. 2). All visits will be separated by a minimum of 48 h. In addition, participants will be required to refrain from exercise, alcohol and caffeine consumption for 24 h before all tests. The familiarisation and baseline testing will consist of the following:

1. 20 km cycle Time Trial (20 km - TT) - During the first (familiarisation) and third visits (baseline test) participants will perform a 20 km - TT on a Velotron® cycle eogometer (RacerMate Inc. Seattle, WA, USA). Participants will complete a warm-up consisting of 5 min of cycling at 60 W. Following a 2-min rest, participants will then be required to complete the 20 km - TT in the quickest possible time. During the time trial, power output measures and time will be concealed from the participants. However, participants will be permitted to monitor their progress through completed distance and will be provided with verbal encouragement during the test.
2. Graded exercise test to exhaustion (GXT) - During the second (familiarisation), fourth, and fifth visits participants will undertake a GXT, for baseline determination of the lactate threshold (LT) and peak aerobic power (W_peak_). This test will be performed on an electronically-braked cycle-ergometer (Lode-excalibur sport, Groningen, the Netherlands) and will consist of 4-min stages separated by 30-s rest periods until exhaustion. The test will start at 60, 90 or 120 W (depending on the participant’s 20 km - TT results) and will be increased by 30 W in each subsequent stage. Capillary blood samples will be taken at rest, after each completed stage, and immediately following exhaustion, and will be analysed by a YSI 2300 STAT Plus system (Yellow Springs, Ohio, USA). During the GXT the LT will be calculated by the modified DMAX method, which is determined by the point on the polynomial regression curve that yields the maximum perpendicular distance to the straight line connecting the first increase in lactate concentration above resting value and the final lactate point [32, 33]. The average of the two GXT tests will be used to individualise exercise intensities, if the difference is no more than 5%, otherwise the highest value will be used.
3. $\overset{\cdotp }{\mathrm{V}}{\mathrm{O}}_{2\mathrm{peak}}$ test - After 5 min rest following the GXT, peak oxygen consumption ($\overset{\cdotp }{\mathrm{V}}{\mathrm{O}}_{2\mathrm{peak}}$) will be measured using a calibrated Quark CPET metabolic system (COSMED, Rome, Italy). Briefly, participants will wear the Cosmed face mask and we will collect VO_2_ at stationary for 2 min, while exercising for 3 min at the intensity of the first stage of GXT (60, 90 or 120 W), and during exercise to exhaustion at 105% of Wpeak measured during the previous GXT. $\overset{\cdotp }{\mathrm{V}}{\mathrm{O}}_{2\mathrm{peak}}$ will be considered the highest value in 1 min obtained during the test. Data from previous studies have shown the $\overset{\cdotp }{\mathrm{V}}{\mathrm{O}}_{2\mathrm{peak}}$ measured this way is not different from that derived from a ramp test [34]. The HIIT phase will commence 48–72 h after the last baseline exercise test.

### Muscle biopsies

Muscle biopsies will be performed on the vastus lateralis muscle of the participants’ dominant leg. Following injection of a local anaesthetic (5 mL, 1% Xylocaine), incisions will be made and the biopsy needle will be inserted. Muscle samples will be collected with manual suction applied [35]. Following collection, the samples (50–200 mg) will be immediately blotted on filter paper to remove excess blood, with a small portion (10–15 mg) immediately processed for the determination of mitochondrial respiration [36]; about 10 mg will also be embedded in Tissue-Tek ® O.C.T. Compound for muscle structure analysis, with the remaining muscle snap frozen in liquid nitrogen before being stored at −80 °C for subsequent analyses.

### Blood sampling

Venous blood samples will be collected through venipuncture or cannulation, immediately after each muscle biopsy. Five mL venous blood will be collected with BD Vacutainer EDTA blood collection tubes (Becton, Dickinson and Company, USA), inverted 6–10 times, centrifuged at 3500 rpm for 10 min at 4 °C, and the resulted supernatant plasma samples will be collected and aliquoted carefully into Eppendorf tubes. The residual blood sample will be saved for DNA extraction. Five mL blood will be collected with BD Vacutainer SST tubes (Becton, Dickinson and Company, USA), left at room temperature for 15 min, centrifuged at 3500 rpm for 10 min at 4 °C, and the resulted supernatant serum samples will be collected and aliquoted carefully into Eppendorf tubes. Three mL blood will be stored in Tempus® Blood RNA tubes (Applied Biosystems, USA) and shaken vigorously for 30 s, and then stored at 20 °C for RNA extraction.

### Acute high intensity interval exercise (HIIE) phase

Following the pre-training baseline muscle biopsy and venous blood sampling, participants will perform a single session of HIIE on an electronically-braked cycle ergometer (Velotron®, Racer Mate Inc., Seattle, USA). The session will consist of eight 2-min intervals performed between the individually-determined pre-training LT power and Wpeak (LT + 40% (Wpeak - LT)), and interspersed with 1-min recovery periods (work-to-rest ratio of 2:1). Muscle biopsies and venous blood samples will be taken immediately after and 3 h post the HIIE to measure muscle and blood biomarkers, including (but not limited to) mitochondrial respiration, transcriptome, protein expression analyses, and enzyme activity (Fig. 2).

### HIIT phase

Participants will be required to train 3 times per week for 4 weeks (12 sessions) (Fig. 2). All training sessions will be completed on an electronically-braked cycle ergometer (Velotron®, Racer Mate Inc., Seattle, USA), and will be preceded by a 5-min warm up at 60 W. Each session will consist of six to fourteen 2-min intervals performed at intensities ranging from the power at their individually-determined LT power plus 40 to 70% of difference (∆) between their individually-determined Wpeak (derived from the baseline GXT test results) and the power at the LT (LT + 40–70% ∆), and interspersed with 1-min recovery periods at a power of 60 W (work-to-rest ratio of 2:1). In order to maintain progression, there will be a different numbers of intervals per session (as shown in Table 1).

**Table 1:** Details of the 4 wk. high-intensity interval training (HIIT) program

Session	Intensity (LT + % of∆)	Number of intervals
1	LT + 40%∆	8
2	LT + 40%∆	9
3	LT + 40%∆	10
4	LT + 50%∆	10
5	LT + 50%∆	12
6	LT + 50%∆	11
7	LT + 60%∆	11
8	LT + 60%∆	12
9	LT + 60%∆	14
10	LT + 70%∆	11
11	LT + 70%∆	9
12	LT + 70%∆	6

∆ = peak aerobic power (W_peak_) – power at lactate threshold (LT); 1 interval consists of 2 min of exercise followed by 1 min of rest. There will also be a 5-min warm-up before each training session and a 5-min cool-down following each training session

### Post-training sampling and tests

At the completion of training, participants will report to the lab four times and the visits will be separated by a minimum of 48 h. During their first visit, a resting muscle biopsy and venous blood sample will be collected (Fig. 2). Before the biopsy, a 48-h control diet will again be given to the participants (as described in the ‘nutrition consultation’ section). At the second and fourth visits, post-training GXTs and $\overset{\cdotp }{\mathrm{V}}{\mathrm{O}}_{2\mathrm{peak}}$ tests will take place at the same time of day as the pre-training to assess changes in $\overset{\cdotp }{\mathrm{V}}{\mathrm{O}}_{2\mathrm{peak}}$ and LT (Fig. 1). The average of the two GXT tests will be used to determine the influence of training on common physiological determinants of endurance performance if the difference is less than 5%. If the difference is more than 5%, the highest value will be used. During the third visit participants will perform a post-training 20 km-TT (Fig. 2).

## Data entry and management

A distributed data entry system has been developed for the gene SMART study using Microsoft software at the Victoria University (R-Drive). Each university will enter the data on its own computer and send it to the data coordinating centre at Victoria University. Victoria University registers collections/data available for reuse in Research Data Australia, the national ANDS registry - http://researchdata.ands.org.au/victoria-university. Victoria University also hosts data.vu.edu.au - an institutional repository that can be used to make appropriate digital collections available for reuse. We will ensure the responsible management of data, materials and records during the project, and that after the project data is retained in a durable format and can be appropriately accessed. Victoria University provides enterprise-grade, secure, storage and backup for safe storage during research and for long term retention. Physical records and material are retained within the university colleges. To mitigate the risks associated with genetic research, all data will be stored anonymously; we will not release gene data to participants, nor release gene data to any third parties. DNA samples will be kept for a minimum of 5 years post publication and will then be discarded.

## Muscle and blood analysis

### Genotyping

Genomic DNA will be extracted from residual blood samples from BD Vacutainer EDTA tubes using the MagSep Blood gDNA kit (0030 451.00, Eppendorf, Hamburg, Germany) or GeneJET Genomic Whole Blood DNA Purification Kit (#K0781 Thermo Scientific, MA, USA). Candidate gene variants will be determined using the TaqMan SNP assay (Applied Biosystems, Thermo Fisher Scientific, CA, USA) by QuantStudio 7 Flex (Applied Biosystems, Thermo Fisher Scientific, CA, USA). Genotyping will be replicated in another independent institute, as previously described [37, 38], to validate the results. A Genome-Wide Association Study (GWAS) approach will also be performed on collected samples (Illumina OmniExpress array chips). Genotyping of genomic DNA will be performed using Human Infinium OmniExpress-24 v1.2 BeadChip, containing a maximally informative set of more than 720,000 tag SNPs. Tag SNPs content is optimized from all 3 HapMap phases. The ~720,000 SNP content also includes non-synonymous SNPs, the MHC region, mitochondrial and Y chromosome SNPs. In addition to these tag SNPs, approx 14,000 highly polymorphic CNV regions are specifically targeted, including segmental duplications and regions in the unSNPable genome. The Illumina® Infinium™ II Assay involves an extremely high volume genotype multiplex reaction using a single bead type and dual colour channel approach. This reaction allows hundreds of thousands of SNPs to be genotyped for a single DNA sample. The Assay accomplishes this high volume multiplex reaction by combining whole genome amplification of a 200 ng quantity of genomic DNA starting material with direct, array-based capture and enzymatic scoring of the SNP loci. Locus discrimination is provided by a combination of sequence-specific hybridization capture and array-based, single-base primer extensions. The Assay will be assessed using the Genotyping Module of Illumina’s Beadstudio software (ver. 2.0).

The dependent variables of the proposed study will be analysed as quantitative traits and where necessary will be normalised by transformation as previously determined. In addition to considering the cohort phenotypes individually we will perform multivariate analyses of phenotypes. For multiple traits that exhibit a multivariate approach we will perform principal components factor analysis (PCFA) to convert phenotypes into a linear combination of independent variables (component traits) that explain a large portion of the overall trait variance. In addition, we will weight the phenotypes included in the PCFA based on heritability estimates calculated in the PLINK program [39]. We will follow-up single gene associations by performing multi-gene signature analysis to identify core genetic pathways involved.

Mitochondrial genome will be sequenced using a Next Generation Sequencing with the Ion Proton Platform (Life Technologies, Thermo Scientific) library sequencing method. Briefly, mitochondrial DNA will be extracted, enriched by long range PCR to produce overlapping fragments covering the entire mitochondrial genome and purified prior to library preparation and NGS. Raw sequence reads will be aligned to the revised Cambridge Reference Sequence (rCRS) using SAMTOOLs to produce Binary Alignment (BAM) files. A custom script will be used to call variants relative to the reference genome. Variants which pass quality control will then be analysed using a logistic regression model in PLINK [39]. Variants with a MAF of <0.01 will be excluded from the final regression model to avoid skewing of results. Common SNPs will be analysed separately using a Fisher’s exact test in Plink v1.09. The regression test will factor in for the covariates age, gender and kinship with RNAfold used to predict secondary structural changes to 12S rRNA. A minimum sequencing depth of 16,000 x coverage will allow us to examine heteroplasmic variants, present in a proportion of sequencing reads.

### Muscle analysis

We will use high throughput muscle analysis for the discovery phase and low throughput for verification, as outlined below.

#### Mitochondrial respiration

Immediately after each resting biopsy, muscle fibres will be separated gently on ice under a binocular microscope in BIOPS solution (2.77 mM CaK2EGTA, 7.23 mM K2EGTA, 5.77 mM Na2ATP, 6.56 mM MgCl26•H2O, 20 mM Taurine, 15 mM Na2Phosphocreatine, 20 mM Imidazole, 0.5 mM Dithiothreitol, and 50 mM MES at PH7.1), and permeabilised in the same solution with 50 μg/ml of saponin (Sigma-Aldrich, St Louis, USA) for 30 min. This will be followed by rinsing the muscle fibres for 3 × 7 min in mitochondrial respiration medium on ice (0.5 mM EGTA, 3 mM MgCl2•6H2O, 60 mM K-lactobionat, 20 mM Taurine, 10 mM KH2PO4, 20 mM Hepes, 110 mM sucrose, and 1 g•L^−1^ bovine serum albumin at pH 7.1). Experiments will be performed on washed muscle fibres under continuous stirring using an oxygraph-2 k respirometer (Oroboros Instruments, Austria), containing 2 mL of mitochondrial respiration medium with additional substrates at 37 °C. The following substrates will be added (final concentration): malate (2 mM) and pyruvate (5 mM) to support electron entry to complex I (CI); MgCl2 (3 mM) and ADP (5 mM) to measure Oxidative phosphorylation (OXPHOS) capacity; Succinate (10 mM) to stimulate CI + II-linked respiration and providing convergent electron input into the Q-junction simultaneously (CI + IIP) [17]. A maximal respiratory capacity is reached when these substrates are present in the respirometer chamber [17]. Cytochrome c (10 μM) will be used to test the integrity of the outer mitochondrial membrane [2]. Electron transfer system capacity (ETS with CI + II-linked substrates, CI + IIE) will be tested by titrating p-trifluoromethoxyphenylhydrazone (FCCP) (steps of 0.5 μM) until maximal noncoupled respiration is reached. Rotenone (0.5 μM) will then be added to block the activity of complex I so that electrons can only enter through complex II (CII). Antimycin (3.75 μM) will be added to block the activity of complex III and to measure the non-mitochondrial respiration. Different ratios (substrate and coupling control ratios) will be calculated from the different titration steps obtained from the protocol used [17].

#### Enzyme assay

Complete enzyme extractions from small pieces of frozen tissues will be done in an ice-cold buffer (50 mg•mL^−1^; containing (in mM): Hepes 5 (pH 8.7), EGTA 1, DTT 1, and 0.1% Triton X-100) using a TissueLyser II (Qiagen, Hilden, Germany). Protein concentration is assessed using the bicinchoninic acid assay. Total activities of cytochrome oxidase (COX), citrate synthase (CS), creatine kinase (CK), adenylate kinase (AK), β-hydroxyacyl-CoA dehydrogenase (HADHA) and lactate dehydrogenase (LDH) will be assayed (30 °C, pH 7.5) using standard spectrophotometric assays.

Activities will be represented as μmol·min^−1^·(g protein)^−1^ or international units (IU). CK isoenzymes will be separated using agarose (1%) gel electrophoresis performed at 250 V for 90 min. Individual isoenzymes will be resolved by incubation of the gels with a coupled enzyme system [40].

#### Western blots

Approximately 10 mg of frozen muscle samples will be homogenised in ice-cold RadioImmunoPrecipitation Assay (RIPA) lysis buffer (50 mM Tris·HCl, pH 7.4, 150 mM NaCl, 0.5% Sodium Deoxycholate, 1% Triton X-100, 0.1% SDS, 1 mM EDTA with protease/phosphatase inhibitors, 1 mM PMSF, 1 g/mL Aprotinin, 1 g/ml Leupeptin, 1 mM Benzamedine, 1 mM Na3VO4, 5 mM Na Pyrophosphate, 1 mM DTT and 1 mM NaF) using a TissueLyser II (Qiagen, Hilden, Germany) for 2 × 1 min at 30 Hz, and rotated for 1 h at 4 °C. Muscle lysates will be stored at −80 °C until further analysis. Total protein content of muscle lysates will be determined using the bicinchoninic acid assay.

Protein extracts will be loaded on sodium dodecyl sulfate polyacrylamide gels, separated for 120 min at 100 V and subsequently transferred to PolyVinyl DiFluoride (PVDF) membranes (Bio-Rad Laboratories, Hercules, USA) using a Bio-Rad blot system for 100 min at 100 V. Thereafter, blots will be blocked for 60 min in 5% milk in tris-buffered saline (TBS) and washed with TBS plus 0.1% Tween at room temperature, followed by incubation with different primary antibodies (including but are not limited to, Citrate Synthase, Cytochrome C, p53, PGC1α, PPARα, total oxphos), UCP3 and etc) overnight at 4 °C. After washing, the membranes will be incubated with the appropriate secondary antibodies for 60 min at room temperature and revealed using a chemiluminescent substrate (Bio-Rad Laboratories, Hercules, USA). Light emission is recorded using ChemiDoc™ MP System (Bio-Rad Laboratories, Hercules, USA) and quantified by image-analysis software (Image Lab, Bio-Rad). Protein content will be normalised to total protein analysis via TGX stain-free gel (Bio-Rad Laboratories, Hercules, USA) [41].

#### RNA extraction, gene expression and whole transcriptome analyses

Total RNA will be extracted from approximately 15 mg of frozen muscle. Cellular membranes will be dissociated in TRIzol® Reagent (Invitrogen, Melbourne, Australia) through TissueLyser II (Qiagen, Hilden, Germany) for 2 × 1 min at 30 Hz. The homogenate will be centrifuged (13,000 RPM for 15 min) and the RNA containing supernatant removed. The homogenate will then be combined with chloroform (Sigma-Aldrich, St Louis, USA) and total tissue RNA is then extracted using the TRIzol protocol in accordance with the manufacturer’s instructions, with the exception of RNA precipitation which will be conducted for a minimum of 2 h at −20 °C in the presence of 10 μL of 5 M sodium chloride. RNA concentration will be quantified spectrophotometrically at 260 nm and purity will be checked using the ratio of its absorbance at 260 and 280 nm using a BioSpectrometer (Eppendorf, Hamburg, Germany). First strand cDNA is then generated from 1 μg of template RNA using the commercially available iScript™ cDNA synthesis kit (Bio-Rad Laboratories, Hercules, USA) using random hexamers and oligo dTs according to the protocol provided with the iScipt cDNA synthesis kit (Bio-Rad Laboratories, Hercules, USA). cDNA will be stored at −20 °C for subsequent analysis. All samples and reverse transcriptase (RT) negative controls will be run together to prevent technical variation. Forward and reverse primers for the target and housekeeping genes will be designed based on NCBI RefSeq using NCBI Primer-BLAST (www.ncbi.nlm.nih.gov/BLAST/). Primers will include but are not limited to, 18 s, B2M, Cyclophilin, GAPDH, TBP, Citrate Synthase, Cytochrome C, p53, PDK4, PGC1α, PPARα, and etc. Specificity of the amplified product will be confirmed by melting point dissociation curves generated by the PCR instrument. The mRNA expression of target and housekeeping genes will be quantified by quantitative real-time RT-PCR (QuantStudio™ 7 Flex Real-Time PCR System (Life Technologies, Thermo Fisher Scientific, Wilmington, DE, USA), using a 5 μL PCR reaction volume and SYBR® Green chemistry (iTaqTM Universal SYBR® Green Supermix, Bio-Rad, Hercules, CA). All samples will be run in duplicate simultaneously with template free controls, using an automated pipetting system (epMotion 5073, Eppendorf, Hamburg, Germany). The following PCR cycling patterns will be used: initial denaturation at 95 °C for 3 min, 40 cycles of 95 °C for 15 s and 60 °C for 60 s.

### Blood analysis

#### Cytokines and other blood markers of trainability

Plasma and serum samples will be analysed to determine cytokines, fatty acids, glucose, insulin and other health-related blood markers. Cytokines will be analysed using Bio-Plex Pro™ Human Cytokine, Chemokine, and Growth Factor Assays (Bio-Rad, California, USA). Glucose will be analysed via a YSI 2300 STAT Plus system (Yellow Springs, Ohio, USA). Insulin will be analysed by using human insulin specific RIA lit (Millipore, Missouri, USA).

#### RNA extraction and whole transcriptome analysis

Total RNA will be isolated from the whole blood collected in the Tempus tubes according to the manufacturer’s instruction (Tempus™ Spin RNA Isolation Kit, Life Technologies, Carlsbad, CA, USA). The purified total RNA will be eluted in 90 μL elution buffer and stored in three aliquots at −80 °C until further analysis. Quality and quantity of isolated RNA will be assessed using the Nandrop Technologies Nanodrop® ND-2000 Spectrophotometer (Wilmington, DE, USA). RNA integrity will be evaluated using the Agilent 2100 Bioanalyzer (Agilent technologies, Santa Clara, USA).

The purified total RNA will be amplified and labelled using the GeneChip® WT PLUS Reagent Kit (Affymetrix, Santa Clara, CA, USA). The labelled samples will then be randomly hybridized to the GeneChip® Human Transcriptome Array 2.0 (Affymetrix, Santa Clara, CA, USA) according to the manufacturer’s recommendations. The GeneChip arrays will be incubated in the GeneChip® Hybridization Oven 645, washed and stained on the GeneChip® Fluidics Station 450 and scanned using the GeneChip® Scanner 3000 7G (Affymetrix, Santa Clara, CA, USA).

## Data analysis

Appropriate statistical tests, with multiple comparisons, will be used for data analysis, using both the SPSS and the R packages. A *p* value will be considered as significant depending on the statistical test and number of tests. Multiple testing corrections will be applied accordingly.

For transcriptome data obtained from the Affymetrix GeneChip, Affymetrix® Expression Console™ (version 1.4.1) will be used to perform initial data QC, visualisation, normalisation and summarisation. Bioconductor packages - “oligo” [42] will be used to analyze Affymatrix GeneChip. CEL files at the probe-level; “rma()” function for background correcting, normalizing and calculating expression, and creating the expression dataset to be used for following analysis; and the “sva” (surrogate variable analysis) package [43] in conjunction with the “f.pvalue()” function, “limma” package [44] and “ComBat()” function for estimating and adjusting for surrogate variables, known batches and other artefacts. “pcaGoPromoter” package [45] will then be used to examine overall data structure after the batch effect removal. Finally, “limma” functions will be used to perform the usual differential expression analysis based on the adjusted data. FDR < 0.05 and a 2-fold change will be considered significant.

## Progress to date

Planning and the development of exercise testing, and exercise trainings began in 2014. Following that, recruitment and data collection was initiated at Victoria University, with 64 participants completing the HIIE and 39 participants completing the HIIT to date. Muscle samples for gene expression and protein abundance are being analysed at Victoria University, while key blood samples are being analysed at the Brighton University centre for blood transcriptome analyses. We anticipate the recruitment of 200 participants across centres by the end of 2020.

## Results and discussion

**Fig. 3:**
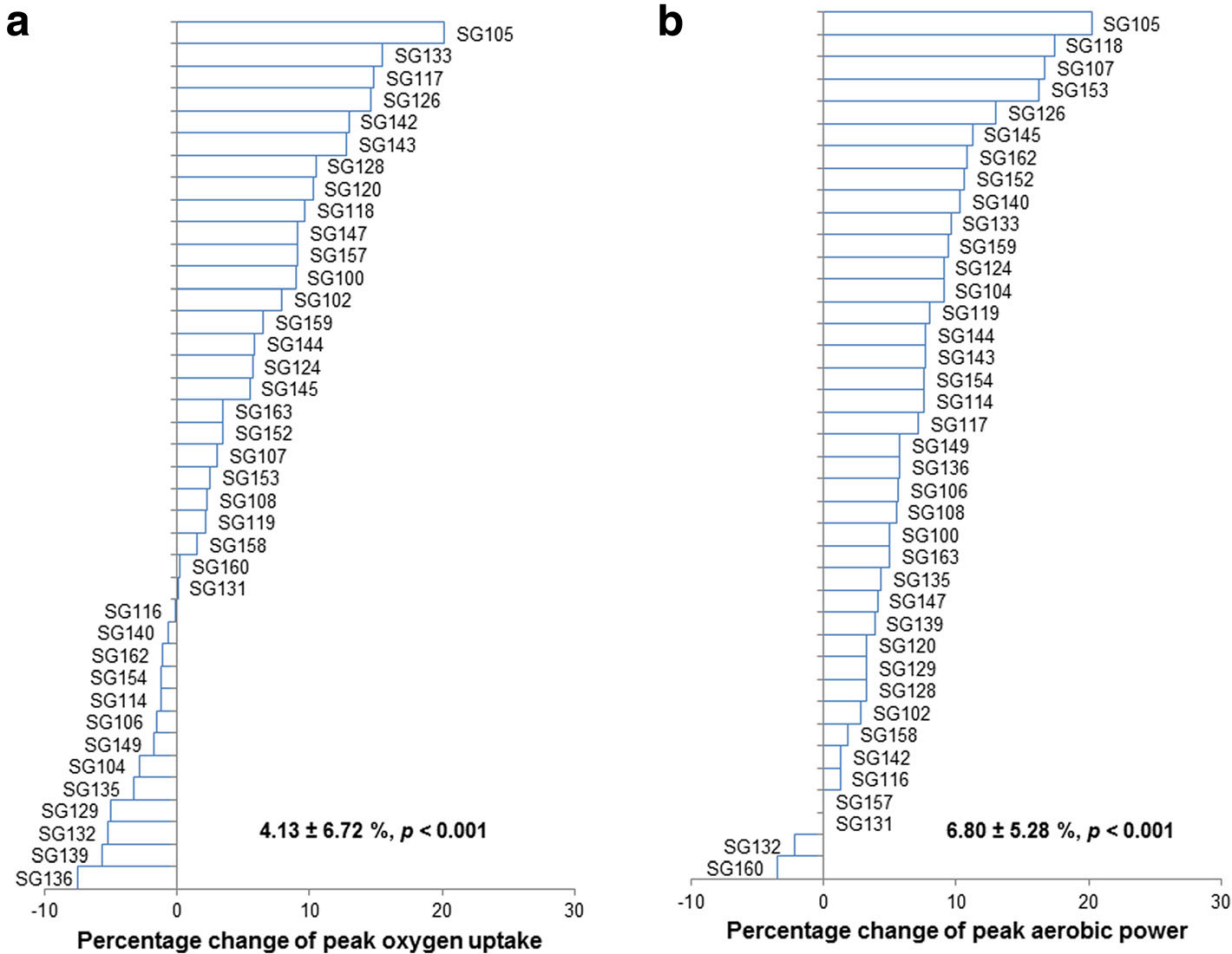
Percentage change of peak oxygen uptake and peak aerobic power after 4 wk. of High Intensity Interval Training (HIIT) as compared with the individual baseline measures (*n* = 39). **a** Change of peak oxygen uptake after HIIT in percentage; **b** Change of peak aerobic power after HIIT in percentage

**Fig. 4:**
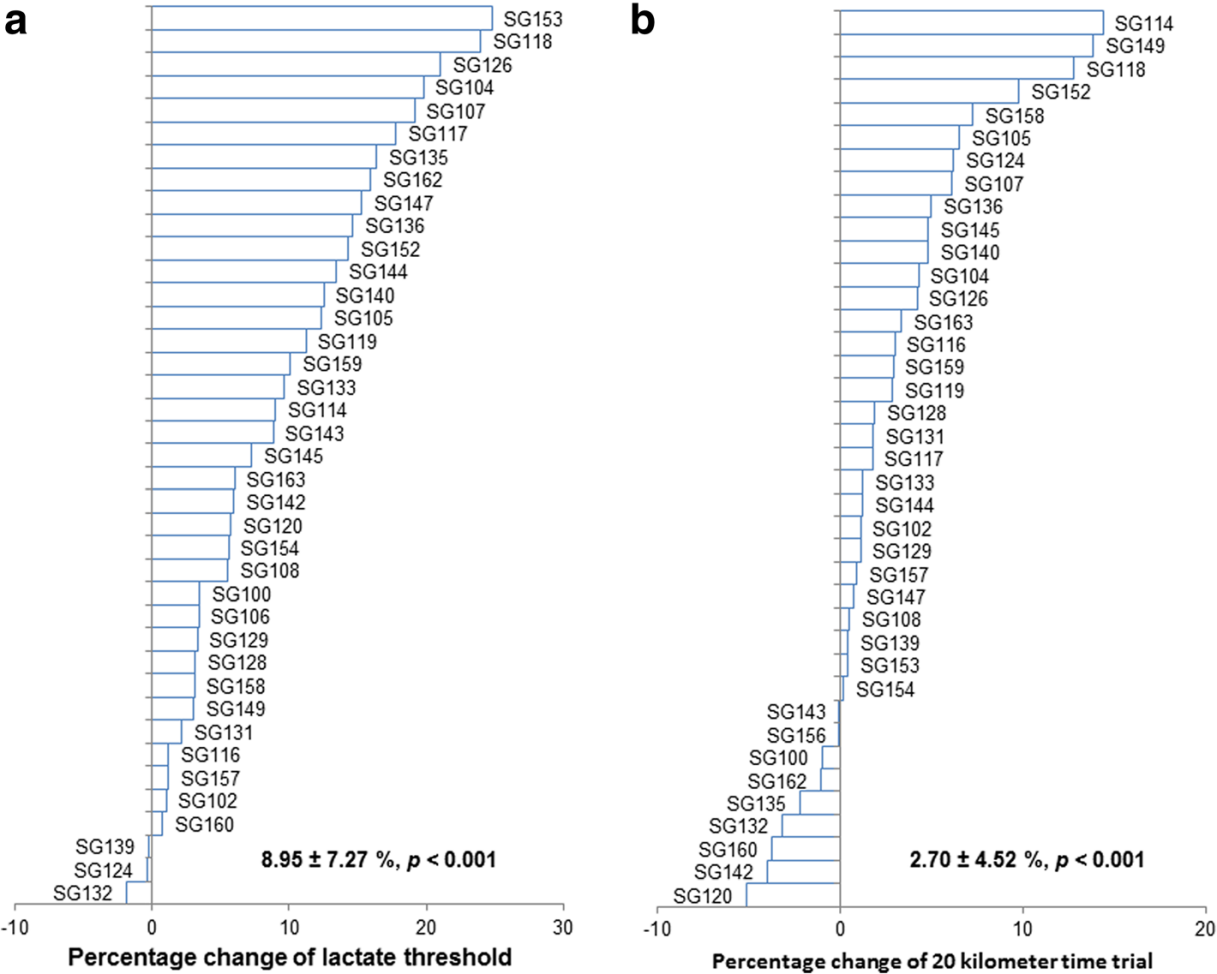
Percentage change of lactate threshold and 20-km time trial after 4 wk. of HIIT as compared with the individual baseline measures (n = 39). **a** Change of lactate threshold after HIIT in percentage; **b** Change of 20-km time trial after HIIT in percentage

Figures 3 and 4 show the individual changes of physiological and performance-related measurements after 4 week HIIT (*n* = 39). As shown in Fig. 3a, the average improvement in $\overset{\cdotp }{\mathrm{V}}{\mathrm{O}}_{2\mathrm{peak}}$ is 3.85% (or 128.5 mL ∙ min^−1^, *p* < 0.001), lower than what was reported in the HERITAGE study (~13% or 393 mL ∙ min^−1^). However, the duration of the HERITAGE study was 20 week (vesus 4 week in the current study), and they recruited non-active or sedentary participants whereas we recruited moderately-trained participants. A recent meta-analysis reported that training-induced gains in the $\overset{\cdotp }{\mathrm{V}}{\mathrm{O}}_{2\mathrm{peak}}$ are generally higher with longer durations and in those with lower baseline $\overset{\cdotp }{\mathrm{V}}{\mathrm{O}}_{2\mathrm{peak}}$ [46] (baseline $\overset{\cdotp }{\mathrm{V}}{\mathrm{O}}_{2\mathrm{peak}}$ in the gene SMART = 3871.82 mL ∙ min^−1^ vs. $\overset{\cdotp }{\mathrm{V}}{\mathrm{O}}_{2\max }$ of 3022.36 mL ∙ min^−1^ in the male subjects of the HERITAGE study [1]). Furthermore, we have previously observed a similar, small percentage increase in $\overset{\cdotp }{\mathrm{V}}{\mathrm{O}}_{2\mathrm{peak}}$ in moderately-trained participants undergoing a similar 4-week HIIT program [47].

The standard deviation for the change in $\overset{\cdotp }{\mathrm{V}}{\mathrm{O}}_{2\mathrm{peak}}$ in the current study is 6.36% or 235.8 mL ∙ min^−1^, similar to the value of 202 mL ∙ min^−1^ in the HERITAGE study [1]. Furthermore, in a recent meta-analysis, changes in $\overset{\cdotp }{\mathrm{V}}{\mathrm{O}}_{2\mathrm{peak}}$ after endurance and HIIT training were reported to range from −455 to 1521 mL ∙ min^−1^ [48]. In the current study, our preliminary results show a range of −280.69 to 629.25 mL ∙ min^−1^. Once again, the lower upper limit can probably be attributed to the relatively short duration of the training program, and relatively high baseline fitness of the participants, in our study compared to many of the studies included in the meta-analysis. Nonetheless, our results support previous research indicating there is considerable individual variability for training-induced changes in $\overset{\cdotp }{\mathrm{V}}{\mathrm{O}}_{2\mathrm{peak}}$.

All other physiological and performance indicators improved significantly after 4wk of HIIT. Figure 3b shows an improvement of 6.80 ± 4.82% W_peak_ (Mean ± SD, *p* < 0.001), which is consistent with the ~8% increase reported when using similar training protocols [35, 36]. The 9.01 ± 6.66% increase in LT after 4 weeks of HIIT (Fig. 4a, Mean ± SD, *p* < 0.001), is also similar to the 7 to 8% increase previously reported in response to similar training [35, 36]. Figure 4b shows that, on average, participants improved their 20 km-TT by 3.34 ± 4.46% (Mean ± SD, *p* < 0.001), consistent with previously reported improvements in this parameter [36]. Thus, our training program was effective to increase common measures of aerobic fitness and endurance performance.

While previous studies regarding the individual responses to exercise mainly focused on $\overset{\cdotp }{\mathrm{V}}{\mathrm{O}}_{2\mathrm{peak}}$ or $\overset{\cdotp }{\mathrm{V}}{\mathrm{O}}_{2\max }$, the gene SMART study is also looking at individual responses in other performance variables, such as W_peak_, LT and 20 km-TT. The standard deviation for the change in these variables with training was 4.82% for W_peak_, 6.66% for LT and 4.46% for 20 km-TT. While no previous study has specifically investigated individual responses of these variables, our mean, SD, range etc., are consistent with other small-scale training studies [36, 47]. Furthermore, these values are consistent with the SD reported for the training-induced change in $\overset{\cdotp }{\mathrm{V}}{\mathrm{O}}_{2\max }$ in the HERITAGE study (~7%; [1]).

**Fig. 5:**
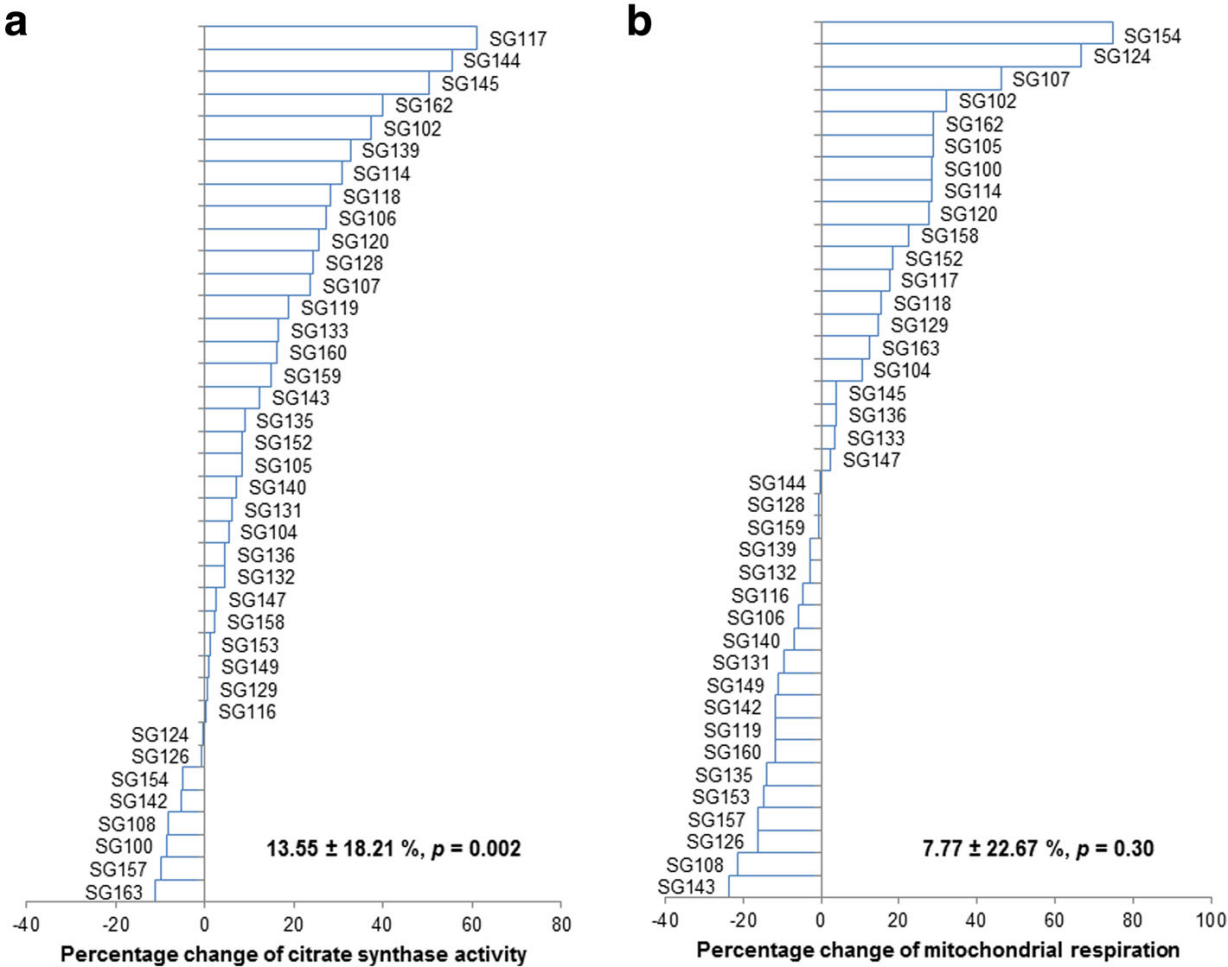
Percentage change of citrate synthase (CS) activity and mitochondrial respiration after 4 wk. of HIIT (n = 39). **a** Change of CS activity after HIIT in percentage; **b** Change of mitochondrial respiration after HIIT in percentage. CS, citrate synthase

An additional novel aspect of the gene SMART study is the investigation of individual responses at the skeletal muscle level. Figure 5a shows an average increase of 13.10 ± 18.02% (Mean ± SD, *p* = 0.004) in citrate synthase activity (a valid indicator of mitochondrial content [49]). The increase is greater than what we have reported when using a similar training program (7.5%, [36]), but less than the 28% increase reported following 6 week of HIIT (10 × 4 min intervals at 90% peak oxygen consumption separated by 2 min rest, 3 days per week [50]). Of note, we have previously reported that training volume appears to be an important determinant of training-induced changes in CS activity [8], and this may help to explain the differences between studies. More importantly, we have observed considerable variability for the training-induced change in CS activity (SD = 18.02%; Fig. 5a). With the gene SMART study, we plan to investigate the possible contribution of genetic factors to this variability.

Similar to what we have reported before [36, 47], we did not observe significant changes in maximal, ADP-stimulated mitochondrial respiration (an indicator of mitochondrial function), after 4 week of HIIT (Fig. 5b, 9.28 ± 21.27%, Mean ± SD, *p* = 0.47). Of note, even though the mean value for maximal ADP-stimulated mitochondrial respiration was not different after four wk. of HIIT, there were some individuals who increased mitochondrial respiration by more than 50%, while others decreased by approximately 20%, which clearly indicates high individual variability in this particular phenotype in response to similar exercise training. The possible contribution of genetic factors to this variability warrants further investigation.

## Conclusions

 Even though the gene SMART study is tightly controlled, we still observe significant variability in both performance (in-vivo) and muscle (in-situ) adaptations to similar training However, while the preliminary data suggest considerable variability in the response of physiological, muscle and performance related factors to 4 weeks of HIIT, more participants are required to allow us to better investigate potential underlying genetic and molecular mechanisms. More participants are being recruited to give us the statistical power to investigate both specific (hypothesis-driven) and non-specific (hypothesis-free) genetic variants as possible predictors, and to follow up these results with the analysis of appropriate molecular/cellular pathways.

## Abbreviations

HIIE: High-intensity interval exercise
HIIT: High-intensity interval training
LT: Lactate threshold
$\overset{\cdotp }{\mathrm{V}}{\mathrm{O}}_{2\mathrm{peak}}$: Peak oxygen uptake
W_peak_: Peak aerobic power
